# Body Appreciation in Light of Psychological, Health- and Weight-Related Variables Among Female Adolescents

**DOI:** 10.5964/ejop.v16i4.2183

**Published:** 2020-11-27

**Authors:** Bettina F. Piko, Annabella Obál, David Mellor

**Affiliations:** a Department of Behavioral Sciences, University of Szeged, Szeged, Hungary; b School of Psychology, Deakin University, Burwood, Victoria, Australia; Glasgow Caledonian University, Glasgow, United Kingdom

**Keywords:** body image, body appreciation, body weight concerns, self-perceived health, female adolescents

## Abstract

Recent research has begun to focus on positive body image and how this can be supported in adolescence. Body appreciation is a key element of positive body image, and has been associated with self-reported health status, weight-related concern, family factors and psychological variables such as self-esteem. In this study we explored these associations among Hungarian adolescent females. Female high school students from two major towns in Csongrád county, Hungary (N = 454; age range from 14 to 20; M = 16.3 years, SD = 1.2) completed questionnaires assessing body appreciation, self-esteem, optimism, life satisfaction and health- and weight-related variables. Analyses revealed that body appreciation was most strongly related to self-esteem, as well as being positively associated with life satisfaction, self-perceived health, being in control of diet, and engagement in sport. Conversely, binge drinking, engaging in slimming behaviors and having eating disorders in the family were negatively associated with body appreciation. These findings provide some indications of factors that might be targetted in health education programs aiming to promote positive body image and to develop resilience against body dissatisfaction in this demographic group. Such programs should also include information of nutrition and media literacy.

In the context of the prevalence of overweight and obesity among children and adolescents having increased over recent decades (NCD Risk Factor Collaboration, 2017), Whitehead et al. (2018) have reported that the fear of weight gain is most prevalent among women aged between 16 and 25. Such weight concerns are associated within a complex web of factors such as body mass index (BMI), negative body image, body dissatisfaction, negative self-concept, and potentially, the development of eating disorders (O’Dea & Dibley, 2014; Whitehead et al., 2018). Therefore, it is important for researchers to focus on developing an understanding of factors relevant to these constructs, particularly among adolescent girls and emerging adult females.

To do this, many studies have focused on the risk factors, correlates and possible consequences of body dissatisfaction and negative body image. For example, body weight perception and lower body satisfaction have been found to predict dieting, unhealthy weight control behaviors and binge eating, and lower levels of physical activity among girls (Kennedy, Schneiderman, & Ramseyer Winter, 2019; Neumark-Sztainer et al., 2006). Negative body image and body dissatisfaction may also play a role in binge drinking and smoking among adolescent girls (Jones, Ramseyer Winter, Pekarek, & Walters, 2018). Further, body image may also contribute to adolescents’ own health, with body dissatisfaction being associated with a higher risk of developing undesirable health-related outcomes (Meland, Haugland, & Breidablik, 2006), including poorer physical competence and increased anxiety level (Dolenc, 2019).

While numerous studies have investigated body dissatisfaction and negative body image, recently, researchers have begun to focus on positive body image. An important element of positive body image is body appreciation, that is, favorable opinions of the body regardless of its actual weight or size, or real or perceived imperfections. Positive body image is more than simply the absence of the elements of negative body image (Dignard & Jarry, 2019) in that it also encompasses a willingness to actively take care of the body’s needs (Tylka & Wood-Barcalow, 2015). Consistent with the proposition that it is not enough to simply remove negative attitudes toward the body, there is also a need to replace them with positive attitudes that may lead to healthy body image (Smolak & Cash, 2011). Thus, some current studies have focused on protective or resilience factors that might assist individuals to resist the strong sociocultural pressures that negatively influence body image and instead, help develop positive body identity (Snapp, Hensley-Choate, & Ryu, 2012). Therefore, in health promotion strategies to facilitate this process we need to strengthen certain skills and attitudes, such as self-esteem, optimism and a positive approach to life (Choate, 2007).

In qualitative studies, body appreciation has been found to be closely related to self-esteem, optimism and life satisfaction (Frisén & Holmqvist, 2010; Wood-Barcalow, Tylka, & Augustus-Horvath, 2010). In quantitative studies with adults, body appreciation has also been found to be positively correlated with self-esteem and positive affect and negatively correlated with negative psychological indicators, such as guilt or shame (Razmus & Razmus, 2017). Several quantitative studies have also found body appreciation to be positively associated with satisfaction with life among youth (Alcaraz-Ibáñez, Cren Chiminazzo, Sicilia, & Teixeira Fernandez, 2017).

Not surprisingly, many of these studies have focused on female populations. In a study of female university students, body appreciation was positively associated with appearance satisfaction, self-esteem and optimism, and negatively associated with BMI (Alleva, Martijn, Veldhuis, & Tylka, 2016). Among college women, it was positively correlated with enjoyment-based physical activity (Homan & Tylka, 2014), health consciousness and health behavior, such as not smoking in a sample of medical students (Dumitrescu, Zetu, Teslaru, Dogaru, & Dogaru, 2008). In a longitudinal study, body appreciation predicted a decrease in dieting, alcohol, and cigarette use, and an increase in physical activity 1 year later (Andrew, Tiggemann, & Clark, 2016).

In addition to psychological and behavioral correlates of youth’s body appreciation and body image, Webb, Rogers, Etzel, and Padro (2018) have found family eating habits and attitudes towards overweight to be important correlates for females. In their study with undergraduate females, family fat talk (e.g., a self-critical talk about being fat) predicted mindful eating via body appreciation. Indeed, disordered eating is often based on intergeneration transmission of attitudes and habits, for example, through learning about appearance standards. In reporting their study of 242 daughter–mother–grandmother triads, Arroyo, Segrin, and Anderson (2017) suggested that direct and indirect maternal communication might facilitate disordered eating, such as dieting or bulimia. Another study found that overweight mothers’ daughters are also more likely to be overweight (Sonneville et al., 2012). However, we know little about how obesity or eating disorders may be associated with adolescents’ body appreciation.

## Present Study

In summary, there is a growing focus on positive body image and constructs such as body appreciation. The current study was conducted in Hungary where the prevalence of obesity and overweight has increased significantly in the past decades, with the nation now occupying a top position not only in European but also in Organisation for Economic Co-operation and Development (OECD) health rankings (OECD, 2017). Further, despite the serious health statistics for conditions in which nutrition and obesity might play a decisive role (Rurik et al., 2016), body image and body appreciation are under-investigated and no effective preventive programs are available for the general population, let alone for adolescents who are in a pivotal life stage for the development of positive body image (Voelker, Reel, & Greenleaf, 2015).

Our study focuses on Hungarian female adolescents who form a vulnerable group of the population not only since they tend to be especially critical about their weight, but also they are receptive to health messages as well. Thus our findings will be useful in informing the development of a health education program. In light of the literature reviewed above, we anticipated that self-esteem, optimism, life satisfaction, good/excellent self-perceived health, diet control and engagement in sport would be positively associated with body appreciation, while BMI, engagement in slimming behaviors, substance use and the presence of overweight or eating disorders in the family would be negatively related to female adolescents’ body appreciation.

## Method

### Participants and Procedure

Data were collected in Szeged and Hódmezővásárhely, two major towns in Csongrád county, Hungary between March and June 2018. The sample consisted of 454 female adolescents (age range from 14 to 20 years; *M* = 16.3, *SD* = 1.2) who studied in different high schools (with a majority of girls, such as specialization of health care or arts) of these two towns. Just over one third (34.4%) of the participants were from grade 9, 33% were from grade 10, 21.4% were from grade 11, and 11.4% were from grade 12. Some classes were unisex and in mixed gender classes only females were asked to participate. Of the 500 students invited to participate, 30 declined. Sixteen others were absent on the day the surveys were administered, giving a response rate of 92%.

Approval to conduct the study was obtained from the Institutional Review Board (IRB) at the University of Szeged, Doctoral School of Education (ref. no.: 6/2017). After gaining the approval, parents were informed about the study and their written consent for their child’s participation was obtained. In addition, students’ informed consent was also obtained. Student participation was voluntary, anonymous, and confidential. A standardized procedure of administration was used in each class: a trained graduate student distributed the questionnaires (paper-and-pencil type) to youth, after briefly explaining the study objectives and giving the necessary instructions.

### Measures

#### Body Appreciation

The Hungarian validated version (Béres, Czeglédi, & Babusa, 2013) of the Body Appreciation Scale - 2 (BAS - 2) comprises 10 items rated on a 5-point Likert-type scale (from 1 = *never*… 5 = *always*; Tylka & Wood-Barcalow, 2015). Using principal component analysis only one component was extracted with a relatively high variance (68.1%) explained. Items measure resondents’ acceptance and favourable opinions of, and respect for their bodies (e.g., “*Despite its imperfections, I still like my body*”). Summed scores were used in the analyses where higher scores reflect higher levels of body appreciation. The Cronbach alpha coefficient of reliability in our sample was excellent (α = .93), similar to the level reported for the original version.

#### Satisfaction With Life

Life satisfaction was measured using the Hungarian validated and adapted version (Martos, Sallay, Désfalvy, Szabó, & Ittzés, 2014) of the Satisfaction With Life Scale (Diener, Emmons, Larsen, & Griffin, 1985). Participants indicated how strongly they agreed with each of the five items using a response format ranging from 1 (*strongly disagree*) to 7 (*strongly agree*). Higher scores reflect higher levels of life satisfaction. The scale was reliable with a Cronbach’s alpha of .83 with the current sample.

#### Optimism

Dispositional optimism was measured using the Hungarian validated version (Béri & Köteles, 2010) of the Life Orientation Test (LOT-R; Scheier & Carver, 1985). The LOT-R consists of ten items (three inverse, plus four filler items that are not scored as part of the scale) assessing generalized expectancies for positive versus negative outcomes. Participants were asked to indicate their level of agreement with each statement using a five-point response scale ranging from 0 (*strongly disagree*) to 4 (*strongly disagree*). This scale was reliable with a Cronbach’s alpha of .74.

#### Self-Esteem

Self-esteem was measured by the Hungarian validated and adapted version (Sallay, Martos, Földvári, Szabó, & Ittzés, 2014) of Rosenberg’s 10-item self-esteem scale (Rosenberg, 1979). Respondents were asked to indicate their level of agreement with each item (five of which are reverse scored) using a scale ranging from 1 (*strongly disagree*) to 4 (*strongly agree*). After reverse scoring the five negatively worded items, responses were summed. Higher scores indicate higher self-esteem. The scale was reliable with a Cronbach’s alpha of .88.

#### Weight-Related Questions

Besides calculating BMI based on the students’ self-reported height and weight, we also asked them about the presence of overweight and eating disorders in the family and whether they are currently on a slimming diet and, whether they take care of their own diet (no/yes).

#### Health Behavior

In terms of other health-related variables, we asked about sporting (no/yes), frequency of smoking, drinking and binge drinking over the last three months (on 6-point scales) as well how they evaluate their own health (self-perceived health in dichotomized form: *fair*/*poor* or *good*/*excellent*).

### Statistical Analysis

SPSS program (Version 22.0.) was used for the analyses. A significance level of .05 was set. Descriptive statistics were calculated and a correlation matrix for the study variables was produced. The contribution of psychological, and weight- and health-related variables in body appreciation was assessed by multiple linear regression models separately, then in combination. Collinearity diagnostics of the multiple linear regression models were also conducted to examine the reliability of the models.

## Results

### Descriptive Statistics and Correlation Matrix for the Study Variables

Table 1 shows the mean scores and standard deviations for the continuous variables and the percentage of participants who responded positively to the categorical variables. The table also summarizes the correlations between variables.

**Table 1:** Descriptive Statistics and Correlation Matrix for the Study Variables (N = 454)

Variable	*M* (*SD*)	%	2	3	4	5	6	7	8	9	10	11	12	13
1. Body appreciation (range: 10—58)	32.69 (9.55)		.74***	.54***	.52***	-.13**	-.13**	-.24***	-.19***	.35***	.18***	-.03	-.11*	.16***
2. Self-esteem (range: 10—40)	25.63 (5.91)		-	.50***	.57***	.01	-.10*	-.19***	-.14**	.19***	.14**	-.03	-.09*	.12*
3. Satisfaction with life (range: 6—35)	22.86 (6.34)		-	-	.50**	-.06	-.10*	-.11*	-.02	.26***	.20***	-.12*	-.08	.15**
4. Optimism (range: 6—30)	19.90 (4.57)		-	-	-	.01	-.09	-.16**	-.14**	.15**	.18***	-.08	-.09*	.11*
5. Body mass index (range: 14.7—43.5)	21.36 (3.93)		-	-	-	-	.23***	-.01	.21***	-.24***	-.06	.05	-.02	.03
6. Overweight in the family (no/yes)		61.1/38.9	-	-	-	-	-	.19***	.04	-.11*	-.04	.03	.01	-.04
7. Eating disorder in the family (no/yes)		93.3/6.7	-	-	-	-	-	-	.14**	-.14**	-.03	-.07	-.08	-.07
8. Slimming behavior (no/yes)		80.8/19.2	-	-	-	-	-	-	-	-.16***	-.16***	-04	-.06	.17***
9. Self-perceived health (fair or poor/good or excellent)		21.1/78.9	-	-	-	-	-	-	-	-	.15***	-.08	-.05	.14**
10. Sporting (no/yes)		39.4/60.6	-	-	-	-	-	-	-	-	-	.06	-.04	.22***
11. Smoking frequency (range: 1—6)	1.54 (1.14)		-	-	-	-	-	-	-	-	-	-	.37***	.02
12. Binge drinking frequency (range:1—6)	1.87 (1.19)		-	-	-	-	-	-	-	-	-	-	-	.01
13. Diet control (no/yes)		28.2/71.8	-	-	-	-	-	-	-	-	-	-	-	-

*Note. r* = Correlation coefficients.

*p < .05. **p < .01. ***p < .001.

First, correlations between body appreciation with psychological and health- and weight-related variables were evaluated. As can be seen in Table 1, there was a close positive association between body appreciation and self-esteem (*r* = .74, *p <* .001). There were also significant positive correlations between body appreciation and life satisfaction (*r* = .54, *p <* .001), dispositional optimism (*r* = .52, *p <* .001), self-perceived health (*r* = .35, *p <* .001), sporting engagement (*r* = .18, *p <* .001), and diet control (*r* = .16, *p <* .001). Conversely, body appreciation was negatively associated with BMI (*r* = -.13, *p <* .01), the presence of overweight (*r* = -.13, *p <* .01), eating disorders in the family (*r* = -.19, *p <* .001), and binge drinking (*r* = -.11, *p <* .05).

In terms of the connections between the psychological and health- and weight-related variables, some significant correlations need also be highlighted. Although BMI did not correlate with any of the other psychological variables, it was associated with the presence of overweight in the family (*r* = .23, *p <* .001) and the tendency to engage in slimming behaviors (*r* = .21, *p <* .001). Those reporting higher BMI rated their own health worse (*r* = -.24, *p <* .001). Diet control showed positive correlations with the psychological variables, sporting engagement, and self-perceived health, but also with dieting to lose weight. Sporting engagement, besides being positively correlated with the psychological variables, was negatively related to slimming behavior. There were negative correlations between slimming behaviors and two psychological variables other than body appreciation, optimism and self-esteem (*r* = -.14, *p <* .01 in each case). Finally, both overweight and eating disorders in the family were negatively correlated with the psychological variables; the correlations were stronger in the latter case (see Table 1).

### Multiple Regression Analysis

Table 2 displays the multiple regression models exploring the contribution of psychological and health- and weight-related variables in female adolescents’ body appreciation, including the collinearity statistics. In the first model, the roles of psychological variables were explored. Self-esteem proved to be the strongest predictor (β = 0.59, *p <* .001). Satisfaction with life played a less role (β = 0.19, *p <* .001) and optimism the least (β = 0.09, *p <* .05). Altogether, these variables accounted for 58 percent of the total variance in body appreciation scores. However, self-esteem accounted for the majority of this (54%).

**Table 2:** Multiple Regression Analysis Assessing the Role of Psychological and Health- and Weight-Related Variables in Female Adolescents’ Body Appreciations With the Collinearity Statistics

Independent variable	Model 1 (B/β/*SE*)	Model 2 (B/β/*SE*)	Model 3 (B/β/*SE*)	Tolerance (VIF)
Psychological factor				
Self-esteem	0.96/0.59/0.07***		0.92/0.57/0.07***	.58 (1.72)
Satisfaction with life	0.28/0.19/0.06***		0.23/0.15/0.06**	.60 (1.66)
Optimism	0.18/0.09/0.08*		0.12/0.06/0.08	.06 (1.67)
Health- and Weight-Related Variable				
Overweight in the family (no/yes)		-0.88/-0.04/0.88	-0.01/-0.01/0.63	.89 (1.12)
Eating disorder in the family (no/yes)		-6.70/-0.18/1.67***	-2.88/-0.08/1.24*	.89 (1.13)
Body mass index (BMI)		-0.06/-0.02/0.11	-0.17/-0.08/0.08*	.85 (1.80)
Slimming behavior (no/yes)		-3.91/-0.16/1.11**	-1.21/-0.05/0.80	.84 (1.19)
Self-perceived health (poor or fair/good or excellent)		5.65/0.24/1.08***	2.99/0.13/0.78***	.82 (1.21)
Smoking frequency		0.59/0.07/0.40	0.47/0.06/0.29	.81 (1.23)
Binge drinking frequency		-0.92/-0.12/0.37*	-0.31/-0.04/0.27	.83 (1.20)
Sporting (no/yes)		2.79/0.14/0.88**	0.26/0.01/0.64	.88 (1.34)
Diet control (no/yes)		1.82/0.09/0.96*	1.10/0.05/0.70	.91 (1.10)
Constant	-1.92	9.94*	-6.36	
*R*^2^	.58***	.22***	.62***	

*Note:* B = unstandardized regression coefficient; β = standardized regression coefficient; *SE* = Standard Error; VIF = Variance Inflation Factor.

*p < .05. **p < .01. ***p < .001.

In the second model, the contributions of health- and weight-related variables were tested. In this model, only 22% of the variance in body appreciation was accounted for. Self-perceived health (β = 0.24, *p <* .001) played the greatest role, but eating disorders in the family (β = -0.18, *p <* .001), dieting to lose weight (β = -0.16, *p <* .001) and binge drinking (β = -0.12, *p <* .05) were also all negatively related to body appreciation, while sporting was weakly but positively associated (β = 0.09, *p <* .05).

In the final model, we entered all predictor variables simultaneously. In this model, self-esteem (β = 0.57, *p <* .001) and life satisfaction (β = 0.15, *p <* .01), self-perceived health (β = 0.13, *p <* .001) and eating disorders in the family (β = -0.08, *p <* .05) remained significant predictors. In addition, BMI became slightly and negatively significant (β = -0.08, *p <* .05). Together, all these variables explained 62% of the total variation in body appreciation scores for this sample of adolescent girls.

The reliability of the models was further examined with Variance Inflation Factor (VIF) indices and tolerance values. The VIF values were within the acceptable VIF range (below 2).

## Discussion

Although body image receives considerable attention in literature, to date more studies have focused on negative body image than on positive aspects, such as body appreciation (Voelker et al., 2015). This is particularly critical in female adolescents for whom developing positive attitudes towards their body can prevent eating disorders and distorted body image later. In health promotion programs, developing and supporting a positive body image can be a key focus. Therefore, in this paper we concentrated on psychological, weight- and health-related contributors to body appreciation in a sample of Hungarian female adolescents. Some of these variables may serve as protective factors, while others may elevate the risk of lower levels of body appreciation.

Our findings support previous suggestions that body appreciation is closely connected to self-esteem: having a higher level of self-esteem may contribute to a positive approach to our body and vice versa (Alleva et al., 2016; Frisén & Holmqvist, 2010; Wood-Barcalow, Tylka, & Augustus-Horvath, 2010). Previous studies also reported a close association between these two variables (Alleva et al., 2016; Razmus & Razmus, 2017). The other two psychological variables played lesser roles in body appreciation than we had expected: satisfaction with life was positively but relatively weakly related, and optimism even less so. Previous studies reported stronger connections with these variables (Alcaraz-Ibáñez et al., 2017; Frisén & Holmqvist, 2010; Wood-Barcalow et al., 2010). While bidirectional associations between body appreciation and these two variables were relatively stronger, in multiple regression analyses their role became weaker. This is likely because self-esteem, as the dominant variable supressed their role in body appreciation. Future research is needed to further clarify these associations and to identify other psychological variables that may contribute to body appreciation among adolescent girls.

Our results also confirmed that body appreciation is closely related to subjective evaluations of health (Meland, Haugland, & Breidablik, 2006). Those participants who perceived their own health to be good or excellent also displayed higher level of body appreciation. Health behavior also played a role, particularly sporting engagement: those who were engaged in sports reported better body appreciation. This is in concordance with previous research findings (e.g., Homan & Tylka, 2014). However, while previous studies also found a relationship between smoking, drinking and body appreciation (Dumitrescu et al., 2008; Jones et al., 2018; Kennedy et al., 2019; Neumark-Sztainer et al., 2006), we found only a slight negative association with binge drinking, with those who engaged in binge drinking tending to be less appreciative of their own body. Although cause-and-effect relationships cannot be identified here, we may hypothesize that those who do not appreciate their own body do not take care of it in an appropriate way. The difference between smoking and binge drinking may be both practical and cultural: While negative impacts of binge drinking can be directly experienced by adolescents, smoking can contribute to longer term health consequences. In addition, female smoking is much more accepted in Hungary than female binge drinking. Another finding also supports interpretation of self care in relation to body image: those who reported higher levels of body appreciation also tended to take care of their diet. This is consonant with other findings (Neumark-Sztainer et al., 2006).

Finally, the role of family has also been implicated in the development of either positive or negative attitudes towards our body (Arroyo et al., 2017; Sonneville et al., 2012; Webb et al., 2018). Our results emphasized the role of eating disorders in the family; the presence of such problems may lead to lower body appreciation among female adolescents. The bivariate relationships suggest that those who reported eating disorders in the family also scored lower on the life satisfaction, self-esteem and optimism scales, and also tended to slim themselves. More research is necessary to gain deeper insight into these associations. In addition, there was a slight but negative correlation between body appreciation and overweight in the family; however, in multivariate analyses it became nonsignificant.

As mentioned above, due to the cross-sectional nature of our research, cause-and-effect relationships cannot be determined from our data. A second limitation is that we relied on self-report for our data. While this is not necessarily a significant issue for the psychological variables, it may have impacted on the reliabilty of our measurement of variables such as BMI, family history of eating problems and engagement in health behaviors. Further, some of the values of correlation and regression coefficients are rather weak. Finally, due to specific cultural context, generalizability of our study may be limited.

There are several contributions of our study to the current literature. Most importantly, confirming the strong association between self-esteem and female adolescents’ body appreciation, our study indicates a need for future research to explore other psychological variables that might also be related to positive body image. In addition, our results also draw the attention to the potential role of family factors (e.g., overweight and eating disorder in the family) in the development of adolescents’ body image. Although qualitative studies can more deeply explore the mechanisms underpinning these associations, our data provide evidence of them. Third, our results also emphasize that sporting and binge drinking may play opposite roles in forming body appreciation among girls.

Furthermore, an important strength of our paper is that our findings highlight the difference in associations of various types of substance use with body image, in that while binge drinking is associated, smoking is not, perhaps due to its social acceptance and its delayed health consequences. Finally, we believe that using a sample of Hungarian adolescents is also a strength since to our best knowledge, body appreciation has not yet been explored in this culture.

### Conclusions

Adolescence is an important life stage when both body image and self-esteem are very vulnerable, particularly among female adolescents. It is important to detect both risk and protective factors that are related to girls’ body appreciation and to help them to develop a healthy attitude towards their body. Our study indicates that 1) there is a close association between female adolescents’ body appreciation and their self-esteem; 2) the association between body appreciation and life statisfaction is also positive, but much weaker; 3) self-perceived health, diet control and engagement in sport are positively related to body appreciation, while binge drinking, dieting to lose weight, and eating disorders in the family may be negative contributors; 4) BMI plays a very limited role in teenager girls’ body appreciation. More research is needed to further clarify the role of the family, and other psychological variables should be included in the studies of this nature.

While body image concerns, eating problems and obesity among adolescents have became targets of health education programs over the last decades in Western Europe, United States, Canada and Australia (Yager & O’Dea, 2010), no similar program has been developed in Hungary. Therefore, research of body image among adolescents – particularly female adolescents in Hungary– should be prioritized in order to inform the development of effective health education programs that focus on positive body image. Such programs should also include information of nutrition and media literacy, strengthening self-esteem and resilience.
